# Next-generation sequencing with a myeloid gene panel in core-binding factor AML showed *KIT* activation loop and *TET2* mutations predictive of outcome

**DOI:** 10.1038/bcj.2016.51

**Published:** 2016-07-08

**Authors:** C Y Cher, G M K Leung, C H Au, T L Chan, E S K Ma, J P Y Sim, H Gill, A K W Lie, R Liang, K F Wong, L L P Siu, C S P Tsui, C C So, H W W Wong, S F Yip, H K K Lee, H S Y Liu, J S M Lau, T H Luk, C K Lau, S Y Lin, Y L Kwong, A Y H Leung

**Affiliations:** 1 Division of Haematology, Department of Medicine, LKS Faculty of Medicine, University of Hong Kong, Hong Kong, China; 2 Department of Pathology, Hong Kong Sanatorium & Hospital, Hong Kong, China; 3 Department of Medicine, Hong Kong Sanatorium & Hospital, Hong Kong, China; 4 Department of Pathology, Queen Elizabeth Hospital, Hong Kong, China; 5 Department of Pathology, Queen Elizabeth Hospital, Hong Kong, China; 6 Department of Medicine, Tuen Mun Hospital, Hong Kong, China; 7 Department of Medicine, Princess Margaret Hospital, Hong Kong, China; 8 Department of Medicine, Pamela Youde Nethersole Eastern Hospital, Hong Kong, China; 9 Department of Medicine, Queen Elizabeth Hospital, Hong Kong, China; 10 Department of Medicine, Tseung Kwan O Hospital, Hong Kong, China; 11 Department of Medicine and Geriatrics, United Christian Hospital, Hong Kong, China

## Abstract

Clinical outcome and mutations of 96 core-binding factor acute myeloid leukemia (AML) patients 18–60 years old were examined. Complete remission (CR) after induction was 94.6%. There was no significant difference in CR, leukemia-free-survival (LFS) and overall survival (OS) between t(8;21) (*N*=67) and inv(16) patients (*N*=29). Univariate analysis showed hematopoietic stem cell transplantation at CR1 as the only clinical parameter associated with superior LFS. Next-generation sequencing based on a myeloid gene panel was performed in 72 patients. Mutations in genes involved in cell signaling were associated with inferior LFS and OS, whereas those in genes involved in DNA methylation were associated with inferior LFS. *KIT* activation loop (AL) mutations occurred in 25 patients, and were associated with inferior LFS (*P*=0.003) and OS (*P*=0.001). *TET2* mutations occurred in 8 patients, and were associated with significantly shorter LFS (*P*=0.015) but not OS. Patients negative for *KIT*-AL and *TET2* mutations (*N*=41) had significantly better LFS (*P*<0.001) and OS (*P*=0.012) than those positive for both or either mutation. Multivariate analysis showed that *KIT*-AL and *TET2* mutations were associated with inferior LFS, whereas age ⩾40 years and marrow blast ⩾70% were associated with inferior OS. These observations provide new insights that may guide better treatment for this AML subtype.

## Introduction

Acute myeloid leukemia (AML) is a group of heterogeneous diseases with distinct clinicopathologic, cytogenetic and genetic characteristics. Conventional therapeutic approaches include induction and consolidation chemotherapy. Patients at high risk of relapse receive allogeneic hematopoietic stem cell transplantation (HSCT) as post-remission therapy. AMLs with translocation involving core-binding factors (CBF) including t(8;21)(q22;q22) and inv(16)(p13;q22) or t(16;16)(p13;q22) constitute a distinct clinicopathologic subtype as defined by the World Health Organization (WHO), and occur in 15–20% of adult patients.^1, 2^ In general, CBF-AML has a superior outcome compared with other cytogenetic subtypes, with a long-term overall survival (OS) of 40–60%.^3, 4, 5, 6, 7^ However, their clinical outcome is highly heterogeneous. Mutations of type III receptor tyrosine kinase *KIT* in the activation loop at exon 17 (D816, N822) and the extracellular domain at exon 8, which are rare in other AMLs, occurred in 12–46% cases of t(8;21) and 9–53% of cases of inv(16).^8, 9, 10, 11, 12, 13, 14, 15^ These mutations may induce ligand-independent KIT activation and are generally associated with a higher risk of relapse and poorer prognosis. The impacts of other gene mutations frequently identified in AML, including *RAS* and *FLT3*, on outcome in these AML are less well defined. With the advent of next-generation sequencing (NGS), the genomic landscape of AML can be examined in detail. In particular, The Cancer Genome Atlas (TCGA) examined the genomic profile of 200 *de novo* AML and provided important genomic information of this disease.^16^ However, the number of CBF-AML in TCGA was limited.

In this study, we examined the clinical outcome of a consecutive cohort of patients with CBF-AML who were treated with a uniform approach and examined their mutation spectrum using NGS. The aim was to define the clinicopathologic characteristics and identify novel gene mutations of prognostic values for the design of better therapeutic strategies in this AML subtype.

## Materials and methods

### Patients

Consecutive patients aged 18–60 years, diagnosed with t(8;21)(q22;q22) or inv(16)(p13;q22) CBF-AML in six regional hospitals in Hong Kong between January 2003 and December 2015, were retrospectively analyzed. The diagnoses were confirmed by fluorescence *in situ* hybridization or reverse transcription-PCR for *RUNX1/RUNX1T1* and *CBFB/MYH11* fusions. All patient records were retrieved and independently reviewed by two investigators. Clinicopathologic features, including age, gender, presenting white cell counts, marrow blast percentage, additional cytogenetic abnormalities, induction and consolidation regimens, HSCT and the source of HSC, were analyzed. The study was approved by the institutional review boards of Hospital Authority (HKU/HA HKW UW14-430; KC/KE-15-0039/ER-3; KW/EX-15-052/85-05; NTWC/CREC/15013) and research ethics committee of Hong Kong Sanatorium & Hospital (REC-2015-02).

### Treatments

Specific chemotherapy regimens are shown in Supplementary Table S1. Induction chemotherapy comprised daunorubicin and cytarabine. Reassessment bone marrow was performed between days 21 and 28 after induction. Consolidation comprised four courses of high-dose cytarabine. Before 2012, two courses of daunorubicin and etoposide were given as consolidation before high-dose cytarabine. Remission and relapse were defined by standard criteria. Salvage chemotherapy included ICE (idarubicin, cytarabine, etoposide), MAC (mitoxantrone, cytarabine), FLAG (fludarabine, cytarabine, granulocyte-colony stimulating factor) and CLARA (clofarabine, cytarabine). Patients who achieved second complete remission (CR2) received the same salvage chemotherapy as consolidation until HSCT or leukemic progression (Supplementary Table S2).

### Allogeneic hematopoietic stem cell transplantation

Indications of HSCT at CR1 for eligible patients included two or more inductions to achieve CR1, additional chromosomal abnormalities and presence of *KIT* mutations. HSCT was recommended for all eligible patients in CR2. Allogeneic HSCT was performed in Queen Mary Hospital. Bu-Cy (busulfan; cyclophosphamide) was employed as myeloablative conditioning, and Flu-Bu (fludarabine, busulfan) and Cy-TBI (total body irradiation) for non-myeloablative conditioning. Patients received standard antimicrobial and graft versus host disease prophylaxis as described previously.^17^ Patients who relapsed after HSCT received one of the salvage regimens aforementioned followed by infusion of mobilized peripheral blood HSC from the original donors.^18^

### Next-generation sequencing

Diagnostic bone marrow samples were collected. DNA was extracted using the QIAamp DNA Blood Mini Kit (Qiagen, Hilden, Germany) and analyzed by MiSeq NGS with the TruSight Myeloid sequencing panel (Illumina, San Diego, CA, USA). The panel targeted 54 genes covering full coding sequence of 15 genes and exonic hot spot for 39 genes (Supplementary Table S3). Workflows of MiSeq sequencing library preparation, variant calling and annotation as well as detection of *FLT3* internal tandem duplication by ITDseek has been previously described.^19^ Complex insertions and deletions were detected by an in-house designed algorithm INDELseek on a Cray XC30 supercomputer (Cray Inc., Seattle, WA, USA).

### Orthogonal validation of detected variants

Mutations with variant allele frequency (VAF) <20% were confirmed by one of the following methods according to the specific gene mutations involved. These included microfluidic PCR using Access Array 48.48 (Fluidigm, South San Francisco, CA, USA) and a different primer panel followed by MiSeq NGS, bi-directional Sanger sequencing or PCR fragment analysis by capillary electrophoresis using ABI 3130xl genetic analyzer (Applied Biosystems, Foster City, CA, USA). Only confirmed variants were analyzed in this study.

### Survival and statistical analyses

Leukemia-free survival (LFS) was defined as the time between first complete remission (CR1) to first relapse. Unless otherwise specified, LFS was censored at the date of the last follow-up or death. Overall survival (OS) was defined as the time between diagnosis and death or the date of the last follow up. Treatment-related mortality was defined as death within 30 days of the last chemotherapy or HSCT. Numerical data were compared using Mann–Whitney *U*-test for nonparametric and Student's *t*-test for parametric parameters. Categorical data were compared using χ^2^ test. Different thresholds for age (increment of 10 years, from 20 to 50 years), white cell counts (increments of 10 × 10^9^/l, from 10 to 100 × 10^9^/l) and marrow blasts percentage (increment of 10%, from 30 to 90%) were tested to identify the optimal cutoffs that best defined LFS and OS. Parameters that fulfilled the proportional hazard assumption with a *P*-value<0.1 in univariate analyses were further evaluated by multivariate analysis with the Cox proportional hazards model. Survival curves were constructed using the Kaplan–Meier method and compared by the log-rank test. All analyses were performed using SPSS (IBM Corp., Armonk, NY, USA). A *P*-value of 0.05 was considered statistically significant.

## Results

### Clinicopathologic characteristics and induction chemotherapy

**Figure 1:**
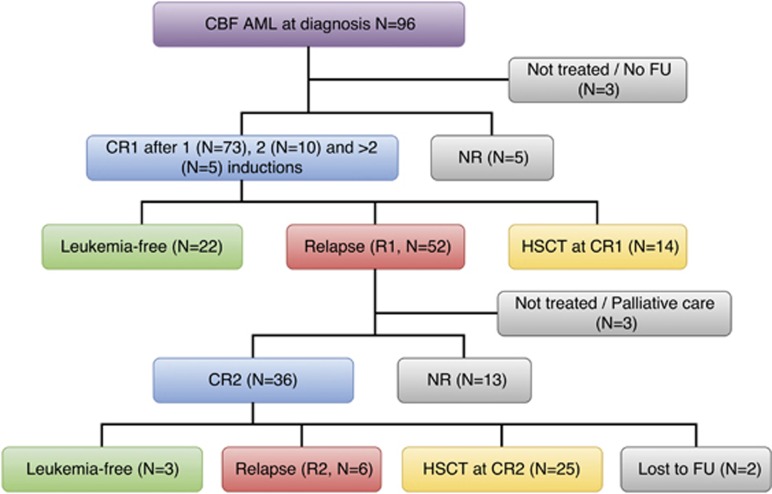
Treatment outcome of 96 CBF-AML patients in this study. Outcome of HSCT has been described in the text. FU, follow-up; NR, nonremission; R1, first relapse; R2, second relapse.

During the study period, 96 patients with CBF-AML were diagnosed. Of these patients, 91 received a standard 7-day regimen of cytarabine (100 mg/m^2^/day) and 3-day regimen of daunorubicin (50 mg/m^2^, *N*=72; 90 mg/m^2^, *N*=18; not specified, *N*=1). One patient received 5:2, and one patient received MAC. Three patients did not receive chemotherapy (Figure 1 and Supplementary Table S2). All 96 patients were included in the survival analysis.

### Treatment outcome

Out of 93 patients, 88 (94.6%) achieved CR1 after one (*N*=73), two (*N*=10), three (*N*=4) and four (*N*=1) courses of induction. Five patients died of refractory leukemia. There was no significant difference in CR rates between AML with t(8;21) and inv(16) (95.5% versus 92.6%, *P*=0.579), and they had similar LFS and OS (Supplementary Figure S1). Fourteen patients received HSCT at CR1, of whom 8 had remained in remission after a median follow-up of 83.8 (44.6–151.3) months. For 74 patients not receiving HSCT at CR1, 22 had remained in remission after a median follow-up of 27.1(4.4–121.6) months. A total of 52 patients had relapsed at a median of 8.9 (1–25.6) months from CR1. Of these patients, 49 received reinduction chemotherapy including MAC (*N*=22), FLAG (*N*=9), ICE (*N*=7), 7:3 (*N*=4), CLARA (*N*=2) and others (*N*=5). Thirty-six patients (73.5%) achieved CR2. Of these patients, 25 underwent allogeneic HSCT at CR2, of whom 13 (52%) had remained in remission after a median follow-up of 67.0 (26.5–137.5) months, 10 (40%) had relapsed at a median of 4.9 (1.6–37.8) months post HSCT and 2 had died of transplant-related mortality. For the other 11 CR2 patients not receiving HSCT, 6 had relapsed at a median of 6.6 (5.4–7.8) months from CR2; 3 had remained in remission 52.6 (2.5–107.4) months from CR2; and 2 had been lost to follow-up (Table 1).

### Mutational analysis

**Figure 2:**
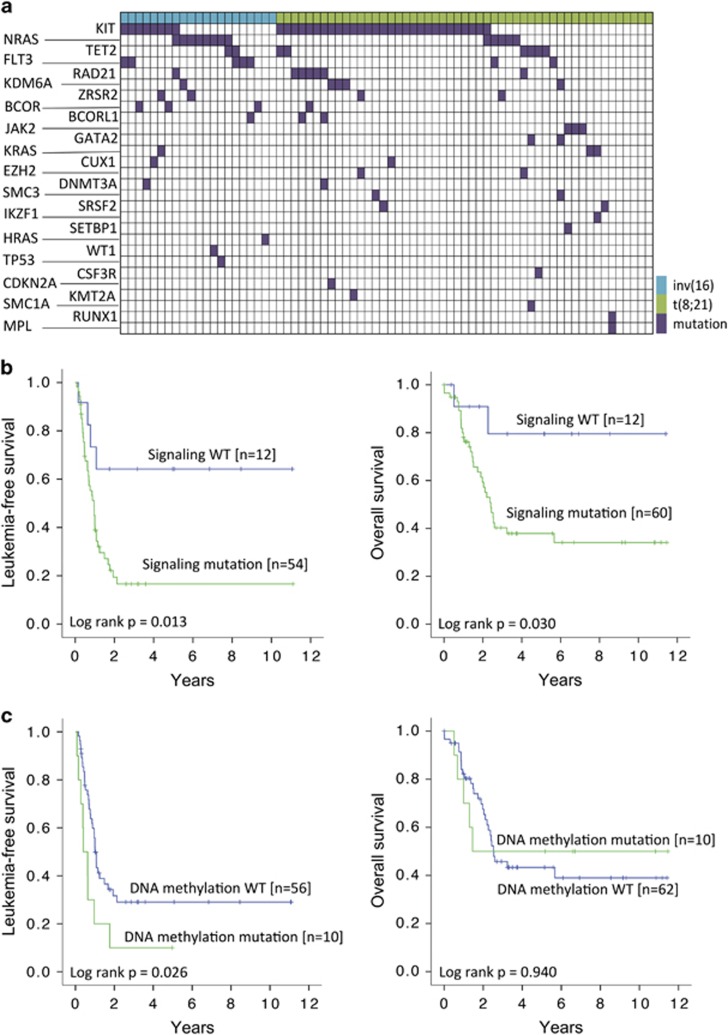
Mutational spectrum and its impact on 72 CBF-AML patients. (**a**) Each column represented data from a single CBF-AML patient. Genetic mutation is colored in purple, t(8;21) in green and inv(16) in blue. (**b**) Survival impacts of mutations of genes involved in cell signaling. (**c**) Survival impacts of mutations of genes involved in DNA methylation.

Genomic studies based on NGS of a myeloid gene panel were carried out in 72 patients, of whom 70 had received induction chemotherapy. When genes were categorized into functional groups, the most common mutations were those involved in cell signaling (*N*=60), chromatin modification (*N*=15), DNA methylation (*N*=10), cohesin complex (*N*=10), RNA splicing (*N*=6), tumor suppression (*N*=5) and transcription (*N*=5) (Supplementary Table S4). Six patients had no mutations and 36 patients showed two or more mutations. The most common recurrent mutations occurred in *KIT* (*N*=37, 51.4%), *RAS* (*HRAS*=1; *KRAS*=3; *NRAS*=13; overall: 23.6%) and *TET2* (*N*=8, 11.1%). Other recurrent mutations included *FLT3* (*N*=7, 9.7%) and *RAD21* (*N*=7, 9.7%) (Figure 2a and Supplementary Figure S2A). The nature of these mutations is shown in Supplementary Table S5.

### Functional group analysis

**Figure 3:**
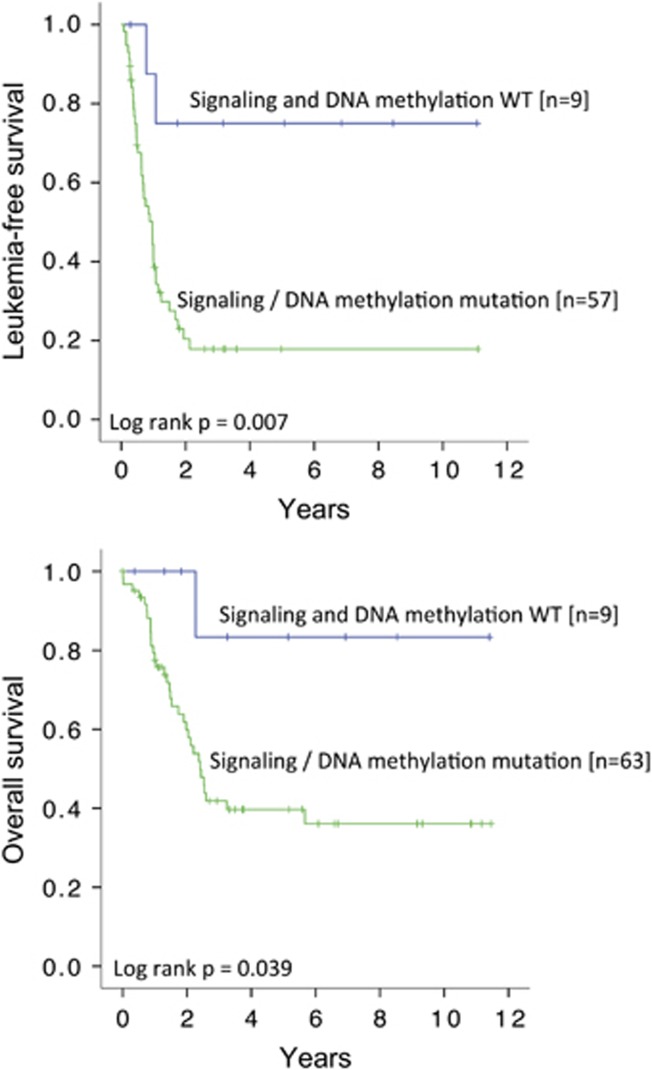
Impacts of gene mutations in cell signaling and DNA methylation on LFS and OS.

To overview the mutation spectrum and to provide mechanistic insights into the pathogenesis of CBF-AML, we evaluated the impact of gene mutations, categorized into specific functional groups, on LFS and OS. Gene mutations involved in molecular pathways of cell signaling were associated with inferior LFS and OS (Figure 2b). Gene mutations involved in DNA methylation were associated with inferior LFS but not OS (Figure 2c). Mutations in other functional groups have no significant impact on either LFS or OS. Importantly, patients negative for gene mutations in both signaling and DNA methylation, compared with those positive for either or both mutations, had significantly superior 10-year LFS (75.0% versus 17.9%, *P*=0.007) and 10-year OS (83.3% versus 36.1%, *P*=0.039) (Figure 3).

### *KIT* mutations

**Figure 4:**
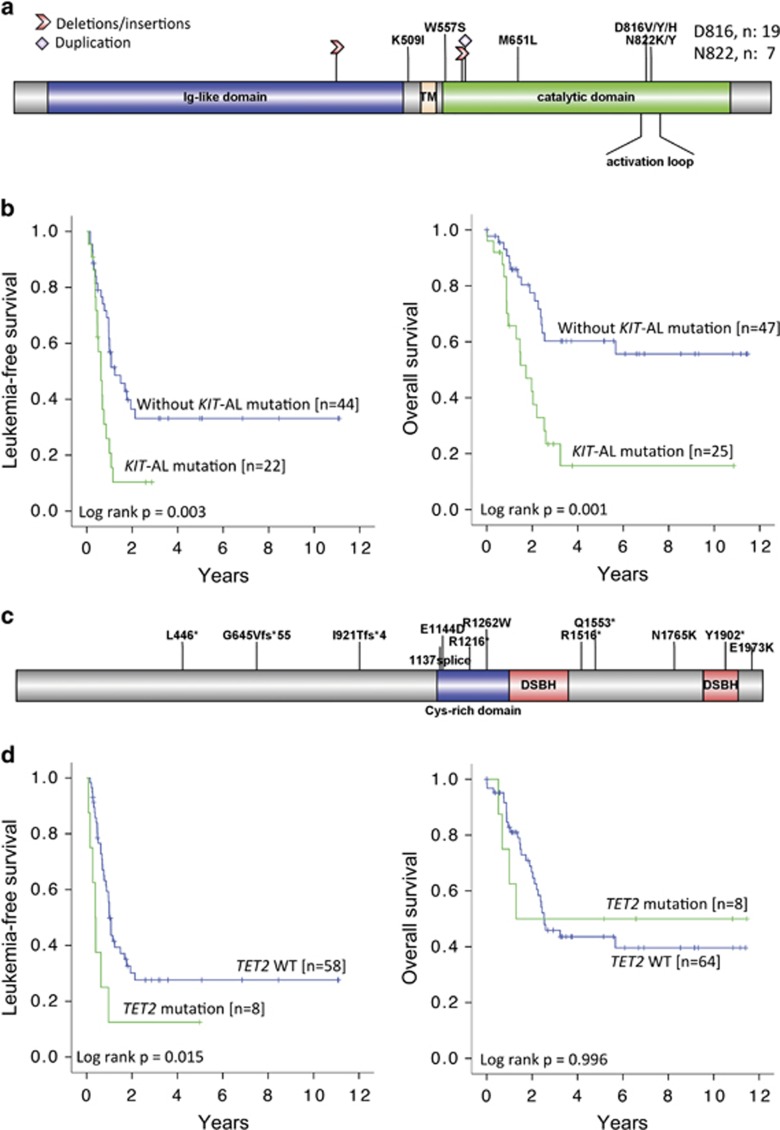
*KIT* and *TET2* mutations in CBF-AML. (**a**) Mutations in *KIT* were concentrated at the AL in exon 17 as shown. Other mutations were scattered in different exons. (**b**) Kaplan–Meier analyses showing that *KIT*-AL mutations were associated with inferior LFS and OS. (**c**) Mutations in *TET2* were scattered throughout the coding sequence. (**d**) Kaplan–Meier analyses showing that *TET2* mutations were associated with inferior LFS but not OS (right panel).

*KIT* mutations were identified in 29 patients with t(8;21) and 8 patients with inv(16). Mutation locations are shown in Figure 4a, being most prevalent in exon 17 (*N*=26, D816 and N822 occurring at the activation loop (AL) of the tyrosine kinase domain) and exon 8 (*N*=13), with others scattered in exons 9, 10, 11 and 12. Seven patients had two or more mutations. AL mutations, when compared with non-AL mutations, were associated with significantly higher median VAF (42% versus 12%, *P*=0.001), comparable median presenting white cell counts (11.7 × 10^9^ versus 20 × 10^9^/l, *P*=0.100) and CR rates (88% versus 97.8%, *P*=0.136), but inferior LFS (*P*= 0.022) and OS (*P*=0.003) (Supplementary Figure S2B). When patients with non-AL mutations were compared with patients without the mutations, LFS and OS were similar (Supplementary Figure S2C). Therefore, patients with AL mutations, when compared with patients having non-AL or no mutations, had significantly inferior LFS (*P*=0.003) and OS (*P*=0.001) (Figure 4b).

### *TET2* mutations

*TET2* mutations, being distributed throughout the coding sequence, were found in eight patients (Figure 4c). All but one patients had VAF >40% (median: 43%, range: 16–53%), and three patients had two or more mutations. Patients with mutations, when compared with patients without mutations, showed a significantly shorter LFS but comparable OS (Figure 4d).

### Other mutations

Mutations in *RAS* (median VAF: 33%, range: 10–77%), *FLT3* (median VAF: 5%, range: 1–53%) and *RAD21* (median VAF: 25%, range 16–46%) did not affect LFS or OS (Supplementary Figures S3A–C).

### *KIT-*AL and *TET2* double mutations

**Figure 5:**
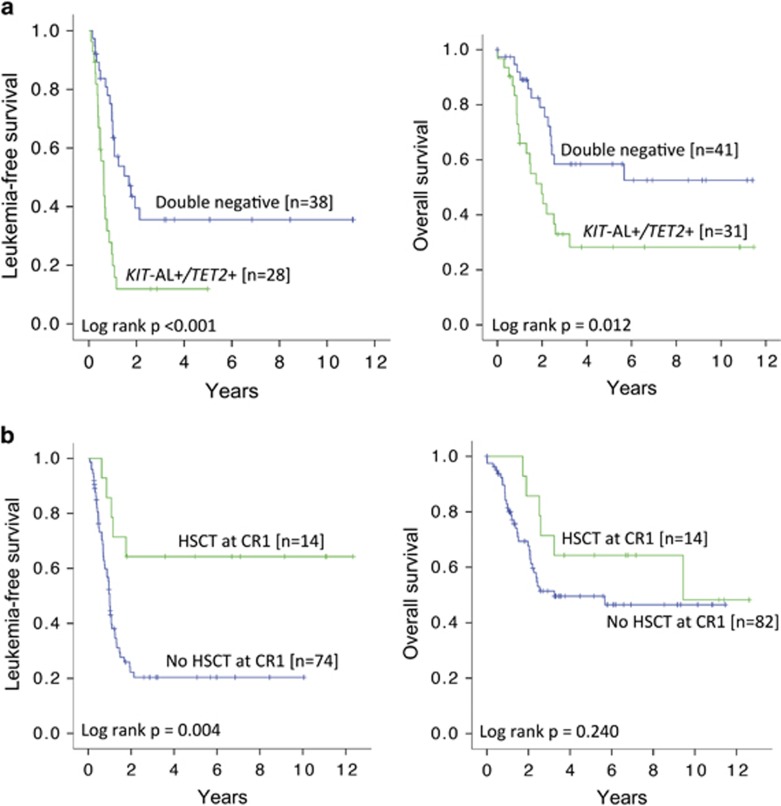
Impacts of *KIT-*AL and *TET2* mutations and HSCT at CR1. (**a**) Kaplan–Meier analyses showing that patients who were negative for both *KIT*-AL and *TET2* mutations had superior LFS and OS compared with those who were positive for either or both of these mutations. (**b**) Kaplan–Meier analyses showing that patients receiving HSCT at CR1 were associated with superior LFS but not OS.

As *KIT*-AL and *TET2* mutations were associated with an inferior outcome, the impact of combined *KIT*-AL and *TET2* mutations was examined. Patients were divided into two groups. Group 1 comprised patients negative for *KIT*-AL mutations (including wild-type *KIT* and non-AL *KIT* mutations) and *TET2* mutations (double negative, *N*=41). Group 2 comprised patients with either one or both of *KIT*-AL and *TET2* mutations (*N*=31). Group 1 compared with group 2 patients had similar CR rates (97.4% versus 90.3%, *P*=0.20), but significantly superior 10-year LFS (35.6% versus 11.9%, *P*<0.001) and 10-year OS (52.6% versus 28.3%, *P*=0.012) (Figure 5a).

### Impact of allogeneic HSCT

Patients undergoing HSCT in CR1, when compared with those without, had significantly better LFS (*P*=0.004) but similar OS (Figure 5b). The impact of HSCT in CR1 for each genetic subgroup was not evaluated because of the small patient number in each category. HSCT from HLA-identical sibling donors or matched unrelated donors showed comparable LFS and OS (Supplementary Figure S4).

### Prognostic factors

Univariate analysis showed that HSCT at CR1 was significantly associated with better LFS. Mutations in cell signaling and DNA methylation as functional groups and *KIT*-AL and *TET2* mutations as individual genes were significantly associated with inferior LFS (Table 2). Age ⩾40 years, presenting white blood cell count ⩾100 × 10^9^/l and marrow blasts ⩾70% were significantly associated with inferior OS. Mutations in cell signaling and *KIT*-AL were also associated with an inferior OS. Multivariate analysis showed that *KIT*-AL and *TET2* mutations were significant adverse factors for LFS, whereas age ⩾40 years and marrow blast ⩾70% were adverse factors for OS. An adverse indicator for OS in univariate analysis notwithstanding, *KIT*-AL mutation did not fulfill the proportional hazard assumption and was excluded from multivariate analysis.

## Discussion

Patients with CBF-AML are clinically heterogeneous despite similar cytogenetic aberrations, implying that secondary mutations might be pathogenetically important. In fact, mutations involving *KIT*, *FLT3* and *RAS* were commonly reported in CBF-AML.^8, 10, 11, 20^ Knock-in mouse models of *RUNX1/RUNX1T1* or *CBFB/MYH11* induced a preleukemia hematopoietic state and required additional mutations for the development of AML,^21, 22^ supporting the proposition of a ‘second-hit' leukemogenic model. With NGS, a recent study recruiting both adult and pediatric patients from two clinical trials has identified mutations in cell signaling, cohesion complex and chromatin modification, being associated with higher risk of relapse.^14^

In this study, we examined the mutation spectrum in CBF-AML and the clinicopathologic features of an exclusively adult patient population who were treated with a uniform algorithm. Mutations of genes involved in cell signaling were associated with significantly inferior LFS and OS, whereas those in DNA methylation were associated with a significantly inferior LFS. Remarkably, patients without any mutations in genes involved in cell signaling and DNA methylation had long-term LFS and OS of ∼80%. The identity of genes in these categories was diverse, with *KIT* and *TET2* being the most common. Other functional groups had no impact on clinical outcome. These results underscore the genetic heterogeneity in CBF-AML, and provide clinical evidence for the importance of concomitant mutations in these leukemias.

In this study, *KIT* mutations were identified in >50% of tested patients, with *KIT*-AL mutations in 35% of them. The prevalence was comparable with those reported based on targeted sequencing.^9, 10^ Importantly, only *KIT*-AL mutations but not other mutations were associated with inferior LFS and OS. Overexpression of *KIT*-AL mutant has been shown to cooperate with *RUNX1-RUNX1T1* or *CBFB-MYH11* to induce AML in mice.^21, 22^ However, *KIT* mutations are known to be unstable during disease evolution, with nearly 50% of cases losing or changing their mutations at relapse.^10^ The mechanistic link between *KIT*-AL mutations and inferior prognosis in CBF-AML would have to be further elucidated.

We also showed for the first time that *TET2* mutations were an adverse prognostic factor in CBF-AML. Earlier studies examining *TET2* mutations with bidirectional Sanger sequencing or 454-based NGS failed to detect any *TET2* mutations in CBF-AML.^23, 24^ Of AML cases examined in TCGA, mutation in *TET2* was shown in only 1 of 7 cases with t(8;21), and none of 11 cases with inv(16).^16^ A more recent study based on RNA-sequencing has shown *TET2* mutations in 3 of 20 cases with t(8;21), but none in 28 cases with inv(16).^15^ In this study, we have shown a similar frequency (6/51) of *TET2* mutations in t(8;21) and an apparently higher frequency (2/21) in inv(16). The scattering of mutations throughout the coding sequence and the lack of hot spot mutations supported the proposition that they were loss-of-function mutations. In all patients but one, *TET2* mutations had VAF >40%, suggesting that the *TET2*-mutant clone was predominant and occurred in a heterozygous state. TET2 functions as a dioxygenase that converts 5 methylcytosine to 5 hydroxymethylcytosine, with a pivotal role in DNA demethylation.^25^ In mice, loss of TET2 has been shown to induce genome-wide enhancer methylation that in a CBF-AML model collaborates to induce an aggressive leukemia.^26, 27^ Further studies are needed to validate the very poor prognostic impact and define the pathogenetic/mechanistic basis of concomitant *KIT*-AL and *TET2* mutations.

In our CBF-AML patients not receiving allogeneic HSCT in CR1, the poor long-term LFS of only 20–30% (Supplementary Figure S5) was comparable with the results from an earlier report on t(8;21) AML from our center^28^ and other reports from Asia,^29, 30^ but inferior to results reported from larger international studies.^31, 32^ It is unclear whether the inferior results of chemotherapy in our patients were because of a different gene mutation spectrum, the small size of this cohort or a difference in treatment regimens. However, the long-term OS was similar to that reported previously,^5^ supporting the notion that relapsed CBF-AML remained sensitive to salvage chemotherapy and patients could still be rescued by allogeneic HSCT.

Because relapsed patients are still salvageable, it remains uncertain whether frontline allogeneic HSCT is needed for CBF-AML. Accordingly, we showed an improved LFS but not OS in our patients receiving allogeneic HSCT in CR1, consistent with results from other studies employing frontline HSCT in CBF-AML, either because of institute policy or guided by minimal residual leukemia.^29, 30, 33^ With improvement in supportive care and advent of reduced-intensity, the indications and potential benefit of upfront HSCT for selected CBF-AML patients, particularly those with unfavorable gene mutation profiles, should be explored.

Our observations have important clinical implications. Patients without *KIT*-AL and *TET2* mutations (‘double-negative' patients) have favorable outcome, suggesting this to be a distinct genetic subgroup that may be curable with chemotherapy. In fact, patients without any mutations involved in cell signaling (which included *KIT*-AL mutations) and DNA methylation (which included *TET2* mutations) had an even more favorable LFS and OS. With the decreasing cost of NGS, a more extensive examination of genetic mutations at diagnosis may become a plausible strategy for prognostication. Furthermore, minimal residual disease monitoring has been shown to predict treatment outcome and provide guidance to HSCT indication in CBF-AML.^33, 34, 35, 36, 37^ Treatment algorithms incorporating mutational profiles and minimal residual disease monitoring should be tested to define whether better risk stratification may be achieved in this AML subtype.

## Figures and Tables

**Table 1:** Clinicopathologic characteristics of 96 patients with CBF-AML

*Features*	*Number (%)*
*Gender*	
Male	53 (55.2%)
Female	43(44.8%)
Age (median, range) (years)	41 (18–60)
Presenting WCC (median, range) (× 10^9^/l)	16.4 (1.6–396.6)
BM blast % (median, range)	50 (20–100)
	
*CBF-AML*	
t(8;21)	67 (69.8%)
inv(16)	29 (30.2%)
	
*Other cytogenetic abnormalities*	
Sole	31 (32.3%)
Additional^a^	65 (67.7%)
*Induction to achieve CR1*^b^	
One	73 (83.0%)
>One	15 (17.0%)
	
*First salvage chemotherapy*	
ICE	1
FLAG	1
MAC	7
Second course of 7+3	6
HSCT	42 (43.8%)
	
*Status at HSCT*	
CR1	14
CR2	25
>CR2	2
R1	1
	
*Source of HSC*	
Sibling	24
Matched unrelated donor	18
No HSCT	54 (56.3%)

Abbreviations: AML, acute myeloid leukemia; BM, bone marrow; CBF, core-binding factor; CR1, first complete remission; CR2, second complete remission; FLAG, fludarabine, cytarabine, granulocyte-colony stimulating factor; HSCT, hematopoietic stem cell transplantation; ICE, idarubicin, cytarabine, etoposide; MAC, mitoxantrone, cytarabine; R1, first relapse; WCC, white cell count; 7:3, cytarabine, daunorubicin.

a Additional chromosomal abnormalities included trisomy-X (*n*=10), trisomy-Y (*n*=24) and others (*n*=31).

b A total of 88 patients achieved CR1.

**Table 2:** Univariate and multivariate analysis of leukemia-free survival (LFS) and overall survival (OS)

	*LFS*					*OS*		
	P*-value*	*Hazard ratio*	*95% CI*		P*-value*	*Hazard ratio*	*95% CI*	
			*Lower*	*Upper*			*Lower*	*Upper*
*Univariate analysis*								
Male gender	0.977	0.99	0.59	1.67	0.448	1.27	0.68	2.38
Age ⩾40 years	0.807	1.07	0.63	1.80	**0.013**	2.31	1.20	4.48
WCC ⩾100 × 10^9^/l	0.557	1.54	0.37	6.42	**0.010**	5.04	1.46	17.37
BM blasts ⩾70%	0.762	0.91	0.48	1.72	**0.010**	2.43	1.23	4.79
>1 induction to CR1	0.584	0.82	0.40	1.68	0.299	1.50	0.70	3.21
HSCT at CR1	**0.006**	0.28	0.11	0.70	0.246	0.60	0.25	1.43
Mutations in signaling	**0.020**	3.42	1.22	9.63	**0.047**	4.26	1.02	17.80
Mutations in methylation	**0.030**	2.26	1.08	4.72	0.940	0.96	0.37	2.50
*KIT*-AL	**0.004**	2.46	1.33	4.55	**0.001**	3.09	1.56	6.12
*TET2*	**0.019**	2.64	1.17	5.97	0.996	1.00	0.35	2.85
*RAS*	0.785	0.91	0.45	1.84	0.804	0.90	0.41	2.00
*FLT3*	0.664	0.80	0.28	2.23	0.966	0.98	0.30	3.19
*RAD21*	0.284	0.57	0.20	1.60	0.406	0.61	0.18	1.98
*KIT*-AL^Neg^ and *TET2*^Neg^	**0.001**	0.35	0.19	0.65	**0.015**	0.43	0.22	0.85
								
*Multivariate analysis*								
Age ⩾40 years	–	–	–	–	**0.013**	2.36	1.19	4.65
WCC ⩾100 × 10^9^/l	–	–	–	–	0.109	2.90	0.79	10.65
BM blasts ⩾70%	–	–	–	–	**0.036**	2.16	1.05	4.44
*KIT*-AL	**0.001**	2.84	1.50	5.36	–	–	–	–
*TET2*	**0.004**	3.43	1.48	7.96	–	–	–	–

Abbreviations: AL, activation loop; BM, bone marrow; CI, confidence interval; CR1, first complete remission; HSCT, hematopoietic stem cell transplantation; WCC, white cell count.

*KIT*-AL^Neg^ and *TET2*^Neg^ was not entered into multivariate analysis, as it would mutually exclude *KIT*-AL and *TET2*. Parameters showing statistical significance are highlighted in bold.
