# Supplementary material for: Next-generation sequencing with a myeloid gene panel in core-binding factor AML showed KIT activation loop and TET2 mutations predictive of outcome
Source: Blood Cancer J. 2016 Jul 8;6(7):e442–. doi: 10.1038/bcj.2016.51 (PMC5030377; doi:10.1038/bcj.2016.51)
Supplement: Supplementary Tables [file bcj201651x1.doc]

Supplementary Table S1. Chemotherapy Regimens used in the study (I)

| Chemotherapy Regimens | | |  |
| --- | --- | --- | --- |
| 7:3 | Cytarabine | 100 mg/m^2^/day | D1-7 |
|  | Daunorubicin | 90 mg/m^2^/day | D1-3 |
|  |  | (50 mg/m^2^/day)^ |  |
|  |  |  |  |
| 5:2 | Etoposide | 100 mg/m^2^/day | D1-5 |
|  | Daunorubicin | 50 mg/m^2^/day | D1-2 |
|  |  |  |  |
| HDAC | Cytarabine | 3 gram/m^2^/dose  (total 4 doses in 2 days) | D1-2 |
|  |  |  |  |
| ICE | Idarubicin | 6 mg/m^2^/day | D1-5 |
|  | Cytarabine | 600 mg/m^2^/day | D1-5 |
|  | Etoposide (VP-16) | 150 mg/m^2^/day | D1-3 |
|  |  |  |  |
| MAC | Cytarabine | 500 mg/m^2^/dose  (total 8 doses in 4 days) | D1-4 |
|  | Mitoxantrone | 12 mg/m^2^/day | D2-4 |
|  |  |  |  |
| FLAG | Fludarabine | 30 mg/m^2^/day | D1-5 |
|  | Cytarabine | 2 gram/m^2^/day | D1-5 |
|  | G-CSF | 300 µg/day | D1-5 |
|  |  |  |  |
| CLARA | Clofarabine | 40 mg/m^2^/day | D1-5 |
|  | Cytarabine | 2 gram/m^2^/day | D1-5 |

^ Patient received 7:3 induction with daunorubicin at 50 mg/m^2^/day before August 2012.

Supplementary Table S2. Chemotherapy Regimens used in the study (II)

| Induction Regimens | Number (%) |
| --- | --- |
| 7:3 | 91 (94.8%) |
| Daunorubicin 50 mg/m^2^ | 72 (79.1%) |
| Daunorubicin 90 mg/m^2^ | 18 (19.8%) |
| Unknown Daunorubicin dose* | 1 (1.1%) |
| MAC | 1 (1.0%) |
| 5:2 | 1 (1.0%) |
| No induction chemotherapy# | 3 (3.1%) |
|  |  |
| Consolidation Regimens |  |
| 5:2 followed by HDAC | 27 (28.1%) |
| HDAC alone | 50 (52.1%) |
| FLAG | 2 (2.1%) |
| MAC | 4 (4.2%) |
| MACE | 1 (1.0%) |
| No consolidation | 12 (12.5%) |
| Defaulted follow-up after initial diagnosis | 2 |
| Defaulted follow-up after CR1 | 2 |
| Directly proceeded to HSCT | 2 |
| Failed to achieve CR1 | 5 |
| Unknown* | 1 |

* Patient received induction in Mainland China. # One patient refused chemotherapy, two patients were lost to follow-up after initial diagnosis. Abbreviations: 7:3, cytarabine, daunorubicin; 5:2, epotoside, daunorubicin; HDAC, high-dose cytarabine; FLAG, fludarabine, cytarabine, G-CSF; MAC, mitoxantrone, cytarabine; MACE, mitoxantrone, cytarabine, etoposide; CLARA, clofarabine, cytarabine; HSCT, hematopoietic stem cell transplantation; CR1, first complete remission.

Supplementary Table S3. Panel of 54 genes analysed by next-generation sequencing of 568 amplicons

| Number | Chromosome | Gene | Regions assessed (exon) | Amplicon count |
| --- | --- | --- | --- | --- |
| 1 | 9 | *ABL1* | 4-6 | 4 |
| 2 | 20 | *ASXL1* | 12 | 19 |
| 3 | X | *ATRX* | 8-10 and 17-31 | 25 |
| 4 | X | *BCOR* | full | 39 |
| 5 | X | *BCORL1* | full | 36 |
| 6 | 7 | *BRAF* | 15 | 1 |
| 7 | 19 | *CALR* | 9 | 1 |
| 8 | 11 | *CBL* | 8 + 9 | 4 |
| 9 | 3 | *CBLB* | 9, 10 | 3 |
| 10 | 19 | *CBLC* | 9, 10 | 2 |
| 11 | 9 | *CDKN2A* | full | 8 |
| 12 | 19 | *CEBPA* | full | 6 |
| 13 | 1 | *CSF3R* | 14 - 17 | 8 |
| 14 | 7 | *CUX1* | full | 48 |
| 15 | 2 | *DNMT3A* | full | 27 |
| 16 | 12 | *ETV6/TEL* | full | 10 |
| 17 | 7 | *EZH2* | full | 21 |
| 18 | 4 | *FBXW7* | 9 + 10 + 11 | 6 |
| 19 | 13 | *FLT3* | 14 + 15 + 20 | 4 |
| 20 | X | *GATA1* | 2 | 2 |
| 21 | 3 | *GATA2* | 2-6 | 10 |
| 22 | 20 | *GNAS* | 8 + 9 | 2 |
| 23 | 11 | *HRAS* | 2 + 3 | 3 |
| 24 | 2 | *IDH1* | 4 | 2 |
| 25 | 15 | *IDH2* | 4 | 1 |
| 26 | 7 | *IKZF1* | full | 11 |
| 27 | 9 | *JAK2* | 12 + 14 | 2 |
| 28 | 19 | *JAK3* | 13 | 1 |
| 29 | X | *KDM6A* | full | 41 |
| 30 | 4 | *KIT* | 2, 8-11, 13 + 17 | 8 |
| 31 | 12 | *KRAS* | 2 + 3 | 3 |
| 32 | 11 | *MLL/KMT2A* | 5-8 | 7 |
| 33 | 1 | *MPL* | 10 | 1 |
| 34 | 3 | *MYD88* | 3-5 | 4 |
| 35 | 9 | *NOTCH1* | 26-28 + 34 | 14 |
| 36 | 5 | *NPM1* | 12 | 1 |
| 37 | 1 | *NRAS* | 2 + 3 | 2 |
| 38 | 4 | *PDGFRA* | 12, 14, 18 | 3 |
| 39 | X | *PHF6* | full | 12 |
| 40 | 10 | *PTEN* | 5 + 7 | 4 |
| 41 | 12 | *PTPN11* | 3 + 13 | 3 |
| 42 | 8 | *RAD21* | full | 17 |
| 43 | 21 | *RUNX1* | full | 13 |
| 44 | 18 | *SETBP1* | 4 (partial) | 1 |
| 45 | 2 | *SF3B1* | 13-16 | 5 |
| 46 | X | *SMC1A* | 2, 11, 16 + 17 | 4 |
| 47 | 10 | *SMC3* | 10, 13, 19, 23, 25 + 28 | 6 |
| 48 | 17 | *SRSF2* | 1 | 2 |
| 49 | X | *STAG2* | full | 40 |
| 50 | 4 | *TET2* | 3-11 | 41 |
| 51 | 17 | *TP53* | 2-11 | 12 |
| 52 | 21 | *U2AF1* | 2 + 6 | 2 |
| 53 | 11 | *WT1* | 7 + 9 | 2 |
| 54 | X | *ZRSR2* | full | 14 |

Supplementary Table S4. Functional groups of mutations detected in our cohort.

| Functional groups | Genes | No. of patient (Individual Gene) | No. of patient (Group) |
| --- | --- | --- | --- |
| Cell signaling | *KIT* | 37 | 60 |
|  | *NRAS* | 13 |  |
|  | *CSF3R* | 1 |  |
|  | *FLT3* | 7 |  |
|  | *HRAS* | 1 |  |
|  | *JAK2* | 3 |  |
|  | *KRAS* | 3 |  |
|  | *MPL* | 1 |  |
| Chromatin modification | *KDM6A* | 5 | 15 |
|  | *EZH2* | 2 |  |
|  | *KMT2A* | 1 |  |
|  | *BCOR* | 4 |  |
|  | *BCORL1* | 3 |  |
| Cohesin complex | *RAD21* | 7 | 10 |
|  | *SMC3* | 2 |  |
|  | *SMC1A* | 1 |  |
| DNA Methylation | *TET2* | 8 | 10 |
|  | *DNMT3A* | 2 |  |
| RNA splicing | *SRSF2* | 2 | 6 |
|  | *ZRSR2* | 4 |  |
| Tumour suppression | *CUX1* | 2 | 5 |
|  | *WT1* | 1 |  |
|  | *TP53* | 1 |  |
|  | *CDKN2A* | 1 |  |
| Transcription | *RUNX1* | 1 | 5 |
|  | *SETBP1* | 1 |  |
|  | *GATA2* | 2 |  |
|  | *IKZF1* | 1 |  |

Supplementary Table S5. Type of mutations found in *KIT, TET2, RAS, FLT3 and RAD21.*

| Gene | Types of mutation | Number of mutation | Percentage |
| --- | --- | --- | --- |
| *KIT* | Missense | 30/45 | 66.7% |
|  | In-frame indels | 14/45 | 31.1% |
|  | Duplication | 1/45 | 2.2% |
| *TET2* | Nonsense | 5/12 | 41.7% |
|  | Missense | 4/12 | 33.3% |
|  | Frameshift indels | 2/12 | 16.7% |
|  | Splicing | 1/12 | 8.3% |
| *NRAS* | Missense | 14/14 | 100.0% |
| *HRAS* | Missense | 1/1 | 100.0% |
| *KRAS* | Missense | 3/3 | 100.0% |
| *FLT3* | Duplication | 5/8 | 62.5% |
|  | In-frame insertion | 2/8 | 25.0% |
|  | Missense | 1/8 | 12.5% |
| *RAD21* | Frameshift indels | 3/7 | 42.9% |
|  | Nonsense | 3/7 | 42.9% |
|  | Missense | 1/7 | 14.3% |
