# Supplementary figures and images for: Next-generation sequencing with a myeloid gene panel in core-binding factor AML showed KIT activation loop and TET2 mutations predictive of outcome
Source: Blood Cancer J. 2016 Jul 8;6(7):e442–. doi: 10.1038/bcj.2016.51 (PMC5030377; doi:10.1038/bcj.2016.51)

Supplementary Figure S1

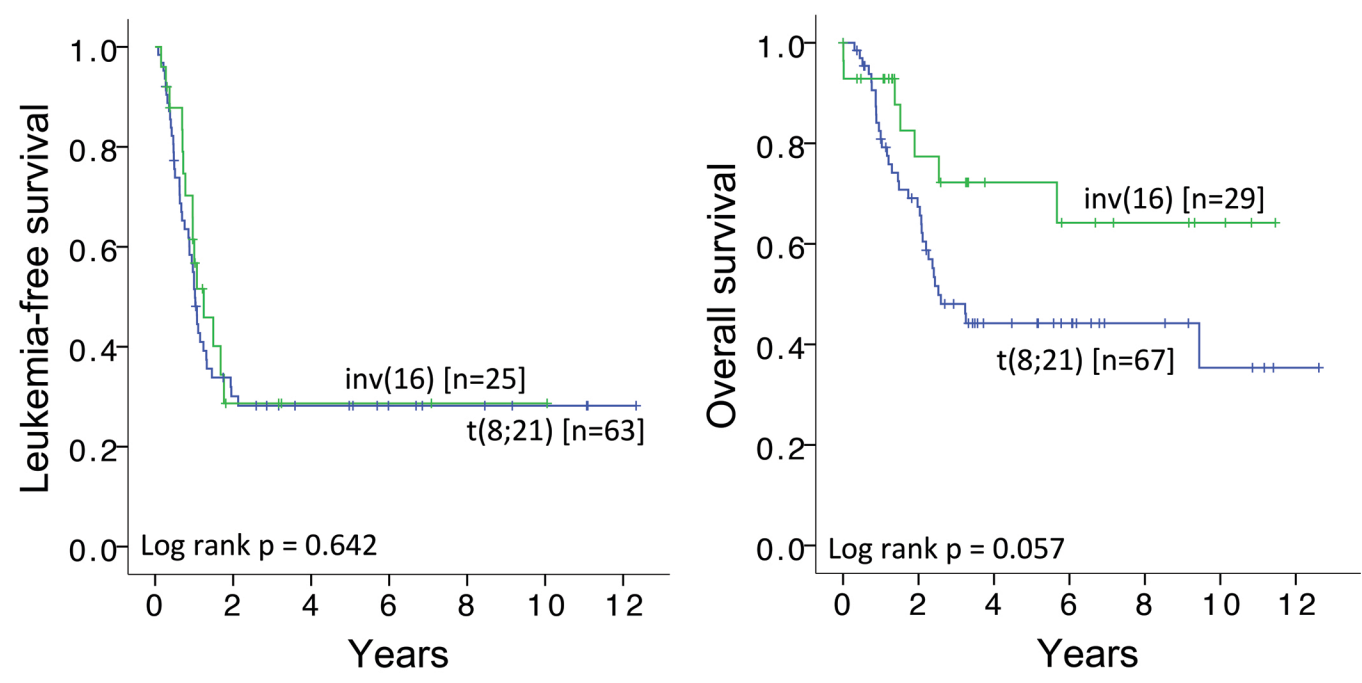

Supplement: Supplementary Figure 1 [file bcj201651x2.pdf]

Supplementary Figure S2

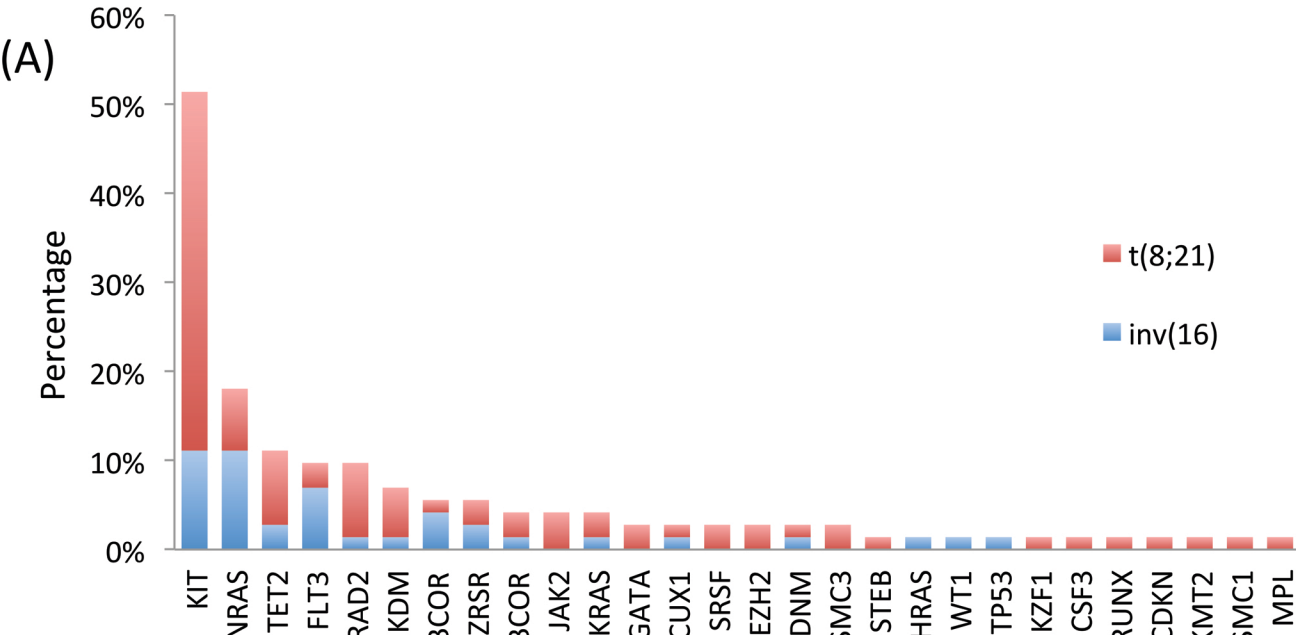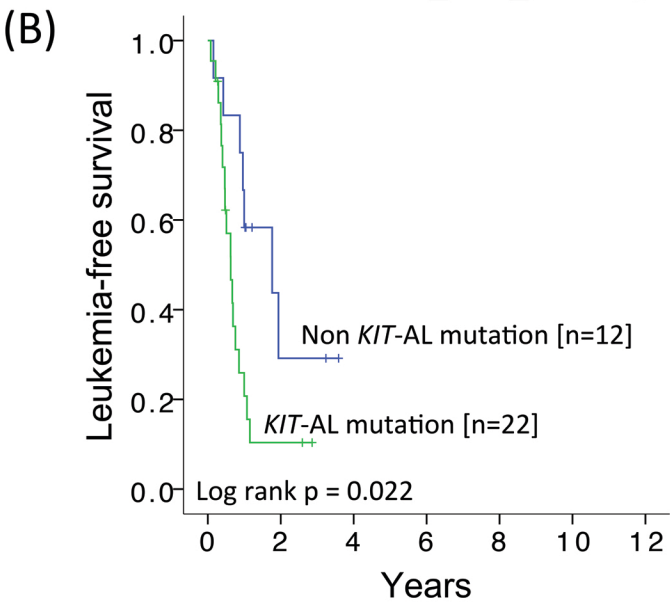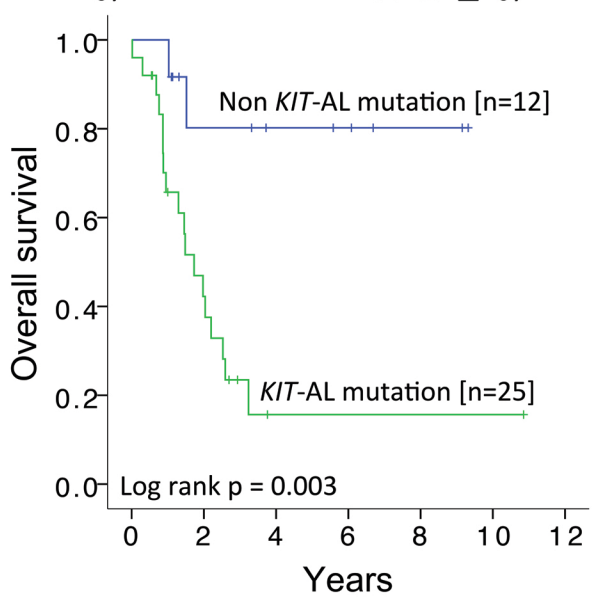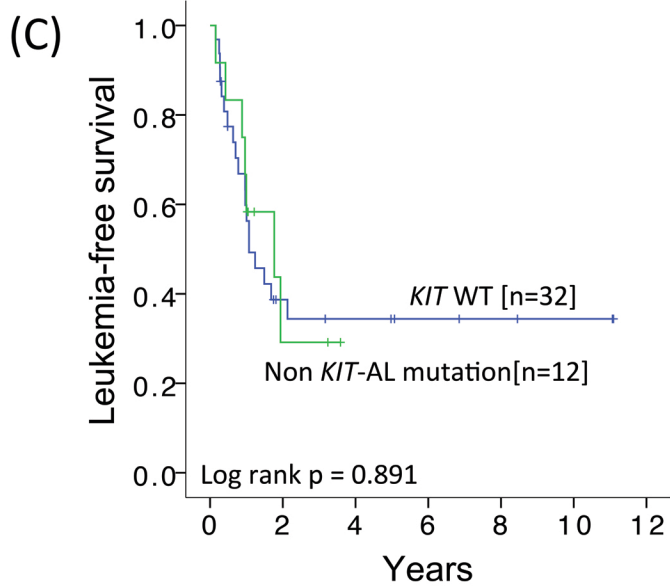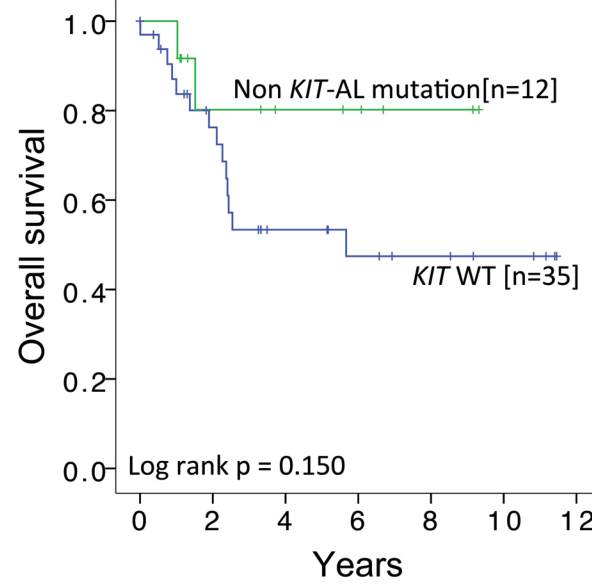

Supplement: Supplementary Figure 2 [file bcj201651x3.pdf]

Supplementary Figure S3

(A)

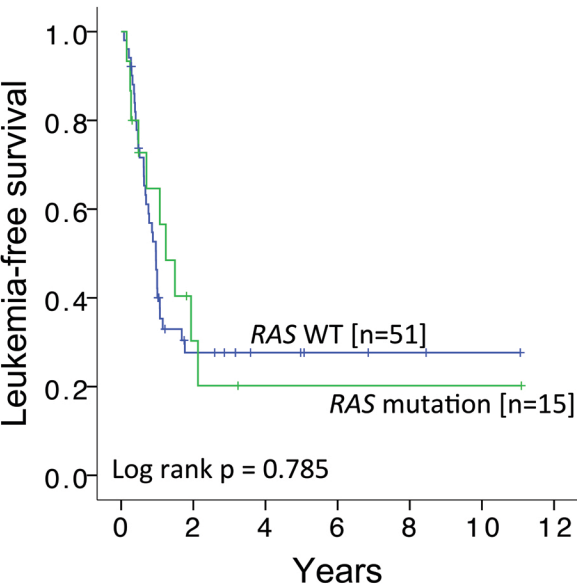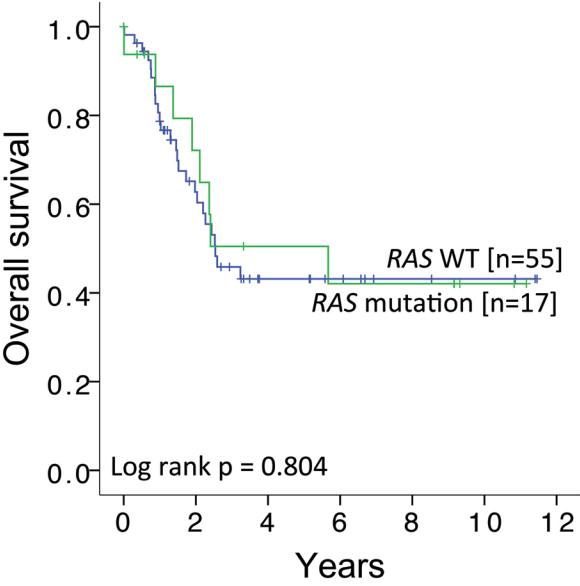

(B)

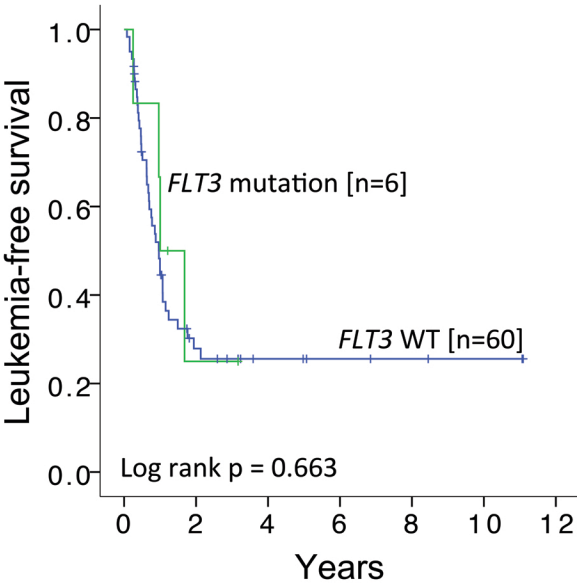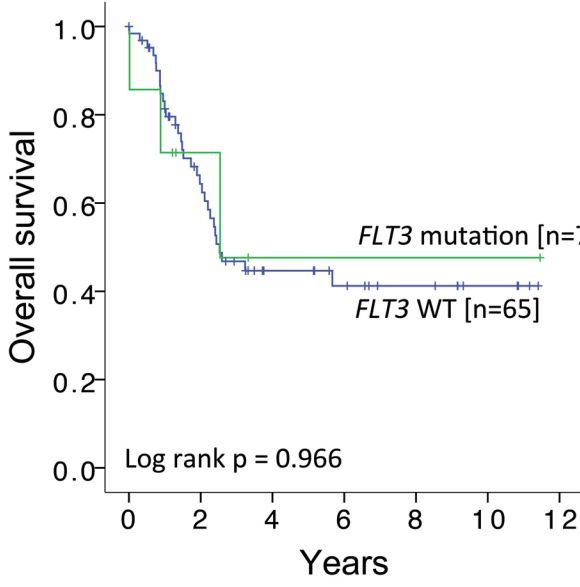

(C)

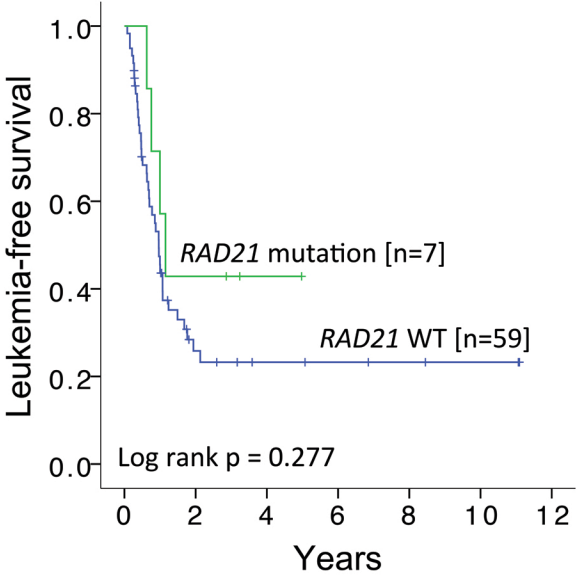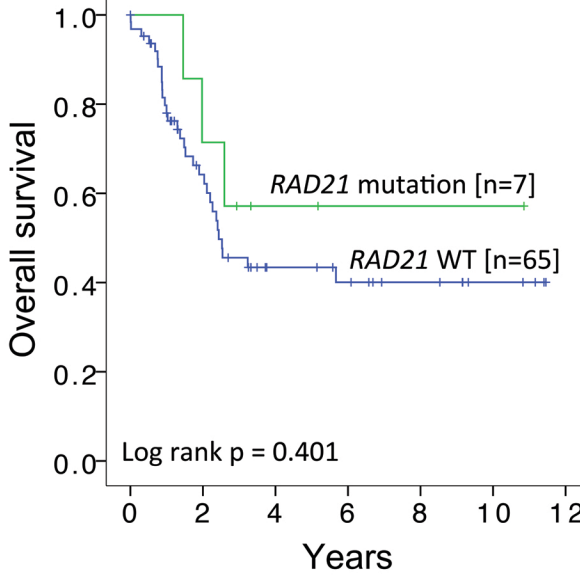

Supplement: Supplementary Figure 3 [file bcj201651x4.pdf]

Supplementary Figure S4

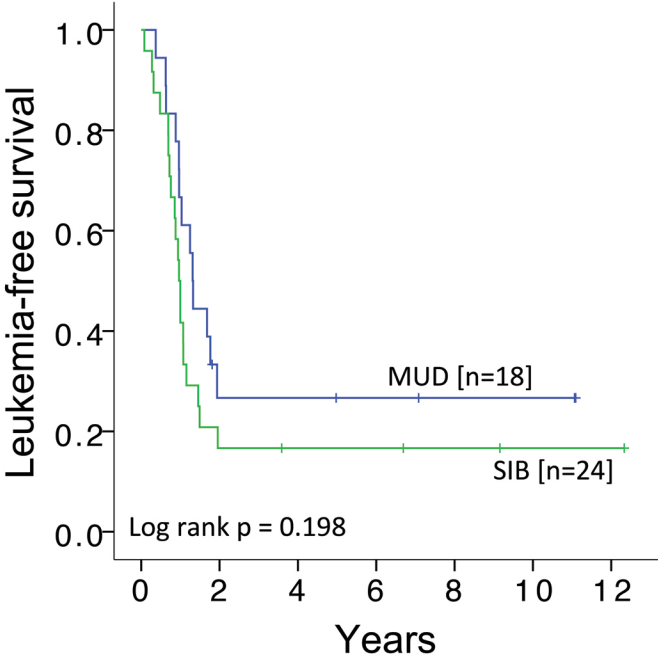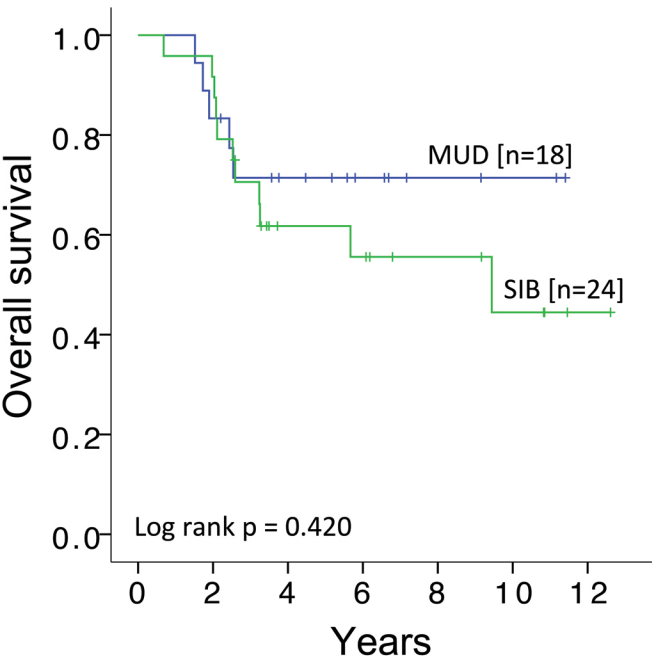

Supplement: Supplementary Figure 4 [file bcj201651x5.pdf]

Supplementary Figure S5

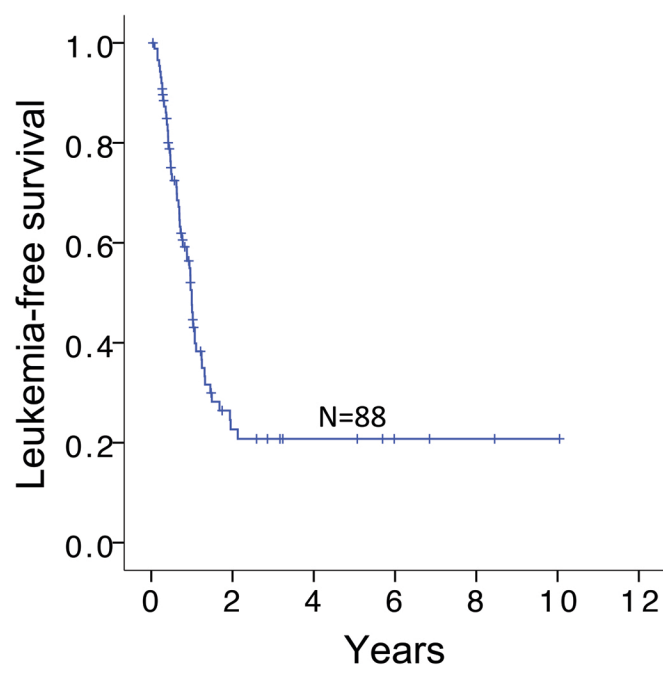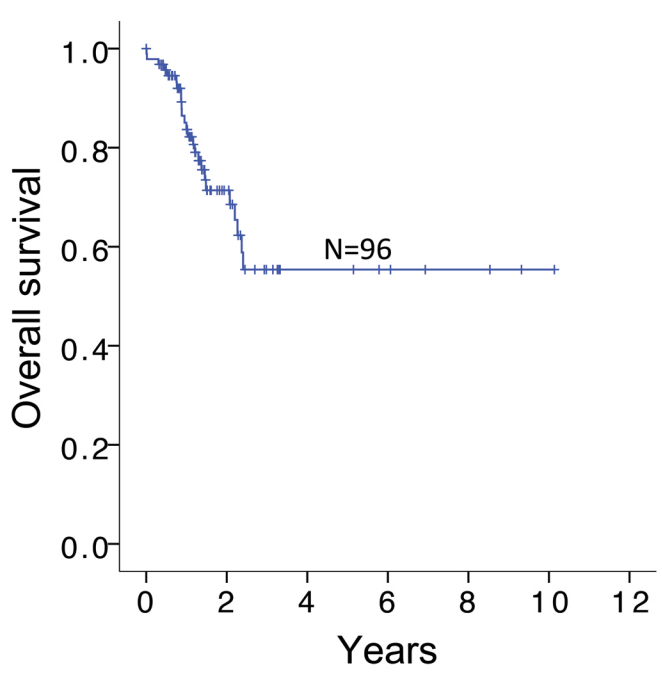

Supplement: Supplementary Figure 5 [file bcj201651x6.pdf]
